# Twenty percent better with 20 micrograms? A qualitative study of psychedelic microdosing self-rapports and discussions on YouTube

**DOI:** 10.1186/s12954-019-0333-3

**Published:** 2019-11-28

**Authors:** Martin Andersson, Anette Kjellgren

**Affiliations:** Dept. of Psychology, Karlstad University, SE-651 88 Karlstad, Sweden

**Keywords:** Microdosing, Psychedelic substances, Self-treatment, Self-optimization, YouTube, Internet support groups, Harm reduction

## Abstract

**Background:**

Psychedelic microdosing is the trending practice of using tiny repeated doses of psychedelic substances to facilitate a range of supposed benefits. With only a few published studies to date, the subject is still under-researched, and more knowledge is warranted. Social media and internet discussion forums have played a vital role in the growing visibility of the microdosing phenomenon, and the present study utilized YouTube contents to improve comprehension of the microdosing practice as well as the social interactions and discussions around microdosing.

**Methods:**

Microdosing self-disclosure in YouTube videos and their following comments were qualitatively analyzed by inductive thematic analysis. Various software was utilized to enable gathering and sorting relevant data.

**Results:**

Microdosing of psychedelic substances, primarily LSD and psilocybin, was used for therapeutic and enhancement purposes, and predominantly beneficial effects were reported. Many different applications and outcomes were discussed, and therapeutic effects for depression appeared especially noteworthy. Intentions for use were recognized as an influencing factor for the progression and outcomes of microdosing. The function of social interactions was mainly to discuss views on the microdosing phenomenon, strategies for optimal results, minimize risks, and share emotional support.

**Conclusions:**

Potentially, microdosing could provide some of the same benefits (for certain conditions) as full-dose interventions with less risk of adverse reactions related to the sometimes intense experiences of higher doses. Microdosing may well also mean additional benefits, as well as risks, through the repeated exposure over extended periods.

## Background

Psychedelic microdosing is the trending practice of using tiny repeated doses of psychedelic substances to facilitate a range of supposed psychophysiological benefits. Major media outlets have noticed microdosing and discoursed it as “the new smart drug,” or “productivity hack” enhancing performance and creativity of professionals in Silicon Valley [e.g., [1–4]]. The origins of psychedelic microdosing are often attributed to James Fadiman and his 2011 book “*The psychedelics explorer guide*”. Dr. Fadiman, in turn, refers his interest in microdosing to information he was entrusted regarding the personal practice of Dr. Albert Hoffman, the inventor of LSD, who allegedly used small doses of LSD for contemplative nature walks [5].

The profound, therapeutic, and sometimes spectacular effects of high or full doses of psychedelics have been extensively researched [e.g., [6–11]], but the practice of using repeated minimal doses of psychedelic substances are still under-researched with only a few published studies to date. Currently, we know of only one double-blinded placebo-controlled microdosing study. In this blinded trial, time perception was investigated, and LSD microdoses were found to produce temporal dilation of supra-second intervals. However, only three doses were administered per subject [12]. Another microdosing study administered measured, but not placebo-controlled, amounts psilocybin-containing truffles exploring the effects of a single microdose on creativity assessed by psychometric scales. Improvements in both divergent and convergent thinking were found [13]. In other microdosing studies, users provided their own substances and effects where evaluated through self rapports, surveys, or psychometric scales [14–17]. Such an approach offers less control; however, these studies had the advantage in exploring the recurrent exposure over time (rather than single or few doses) usually intrinsic to the psychedelic microdosing practice. They also looked more broadly on multiple variables of potential microdosing effects and found improvements for mood, creativity, and depression, increased open-mindedness, less dysfunctional attitudes, stress, and distractibility [14–17].

Worth mentioning is also the research during the 1940–1970s exploring minimum effective dose for psychedelic substances. One placebo-controlled study of low-dose LSD administration showed both physiological (skin galvanic response) and psychological effects (alertness, sociability, and hedonic tone) at 7 μg LSD [18]. The same study found pronounced psychological effects at 20 μg LSD, including euphoria, hypomania, and distractibility. Both the 20 μg and 7 μg doses produced shifts in affect and energy levels during the experiment, and several subjects described this as a “rebirth” or “cleansing” process. Contrastingly, other early studies did not find any subjective effects at 30 μg LSD [19, 20]. One low-dose psilocybin study found that 2 mg doses were recognized by subjects [21], and another study demonstrated perceptible effects with as low doses as 6 μg per kilogram body weight [22]. Our awareness of the abovementioned early/older studies is credited to Torsten Passie and his recent book on microdosing [23]. In this book, Dr. Passie also puts forward definitions of dose ranges and differentiates between microdosing and slightly higher “mini-doses” and proposes the latter as the likely culprit of perceptible acute effects commonly reported by microdosing proponents.

Social media and internet discussion forums have played a substantial role in the growing visibility of the microdosing phenomenon [16]. In recent years, the microdosing forum of Reddit.com has annually doubled the number of subscribers [15] (currently around 55,000). Our interest in microdosing spurred in part from our latest study of drug discussion forums, investigating self-treatment of cluster headaches and migraines [24]. One of the findings was how psychedelic microdosing reportedly functioned as a successful self-treatment for headache disorders. In addition to alleviation of headaches, some of the microdosers mentioned further beneficial effects like increased optimism, creativity, and self-awareness. Our research team has published several studies based on data from online drug discussion forums. This exploration has provided insights into the motivation and views of drug users as well as the effects and risks of emerging psychoactive substances [25–28].

Self-reporting and sharing of drug experiences in online text-based drug forums have proved to be an expedient source of qualitative data for research, especially regarding new or under-researched substances and drug phenomena [e.g., [24–31]]. Obtaining data from social media is cost-effective and quick and can provide information hard to uncover from other sources like poison center calls, survey data, or clinical research. However, one weakness of the results from drug forum data might be a tilt in the demographic profile towards a more knowledgeable or specialized type of drug user than average, which possibly reduces the generalizability of results. We wanted to further develop our method to obtain broader data, both demographically and in terms of content. Noticing the growing range of drug information, user stories, or disclosure of drug use published and discussed via YouTube.com, we identified a need to develop our methodology to address this reality.

YouTube rapidly expands and has become the second most used website (and search engine) in the world [32]. In so doing, it has developed a unique role as a major repository of popular culture as well as an extensive source of information on almost any conceivable subject. It is hard to overstate the enormous impact on-demand online video streaming exerts on the information landscape and how we consume, produce, and exchange information in the current society. Consequently, YouTube is an important arena for deepening awareness of how attitudes, culture, and knowledge of drug use are formed and mediated today. The combination of audio, video, text-based comments, metadata, and the many interactive functions of YouTube provides multilayered and rich data freely available for researchers. Albeit, this enormous expanse of data also means challenges in developing methods to congregate, analyze, and present the results. The present study is an attempt at doing so by utilizing a host of software to facilitate gathering and sorting relevant data on psychedelic microdosing for qualitative analysis.

With this research, we hope to add to previous microdosing studies that were either based on limited demographics [12, 13, 16] or had exclusion criteria for participants with various diagnoses [12]. Our approach does not exclude any type of microdosing user or incentives for use. We also add to previous microdosing research by including interactions and discussions among microdosers via the comments sections and other interactive functions of the YouTube platform.

## Aim

The aim of the study was to improve knowledge of the recent and contemporary phenomenon of microdosing psychedelics as it is presented and discussed on the popular video-sharing platform YouTube.com. This study was also conducted as a first step in developing a method for using YouTube as a data source for research in our field.

## Methods

### Data collection and procedure

Data for the present study was achieved by using specialized software and a YouTube API key. To obtain an initial dataset of relevant YouTube videos, a query search with the single keyword “microdosing psychedelics” was performed using the software Webometric analyst 19 September 2018. The keyword was selected to be unconfined and not limited to a particular microdosing purpose or substance. Our initial search generated 84 videos, which were manually screened and curated. Nine videos were removed for being irrelevant (not microdosing), duplicate uploads, or not in English. The remaining 75 videos were further examined in order to (1) get a comprehensive overview and (2) select all videos containing self-rapports (first-hand accounts) of microdosing a psychedelic substance. Step two generated 32 videos with self-rapports that were included in the final dataset used for the qualitative analysis. The 32 videos were downloaded with their subsequent comments section by using the browser add-on Ncapture. Also, transcriptions (auto-generated) for the 32 videos were obtained via the website https://www.diycaptions.com. The transcripts were then manually revised in conjunction with a screening of the videos, providing the researcher with an opportunity for in-depth acquaintance with the material. The 32 video IDs were then used as input for the software YouTube statistics to obtain data on views, ratings, and comments. Also, the channel IDs were used as input for Webometric analysts to obtain data on subscribers and channel view counts. Finally, the comments, videos, and transcripts were imported to Nvivo11 for qualitative analysis.

### Sample

The 32 videos contained self-rapports from 34 individuals (24 male, 10 female). From the comments, we included statements and self-rapports from 198 users. However, gender could not be assessed since an anonymous alias was the only available information on commenters. The total length of the included videos was 15 h 7 s, and the average length was 36 m 5 s. The transcriptions of the videos consisted of 142,769 words or 763,307 characters (including spaces). The comments consisted of 4507 entries. At the date of data collection, the total views of the included videos were 1,229,336 (average views 38,416.75). Total likes were 26,152 (average 817.25), total dislikes 947 (average 29.59), and total raters 27,099 (average 846.84). The videos were uploaded by 25 channels (or senders) with a total of 4,501,935 subscribers and were posted in eight different YouTube categories.

### Analysis

The transcripts and the collected comments of the 32 videos were qualitatively analyzed using inductive thematic analysis, according to Braun and Clarke [33]. No initial hypothesis was used; the investigation was data-driven and coded fully inductively, albeit defined by the scope of the study. Since the videos and comments also contained, for this study, irrelevant or of topic content, we included exclusively statements based on first-hand experiences of psychedelic microdosing. All statements constituting mere second-hand information were ignored. Also, discussions about the procurement, cultivation, or production of substances were considered out of scope and excluded. One challenge was to decide inclusion when general viewpoints (rather than overt self rapports) on microdosing were discussed. The guiding principle used was to include general reflections in the analysis only when the same individual also had direct personal experiences of the phenomenon discussed. For instance, general reflections on how trauma can affect the nervous system and health, and how microdosing can play a role in a healing process was included only if the person also had experiences of the same.

The coding was conducted on a semantic or explicit level within a realist epistemology; interpretations of latent content were kept at a minimal. The checklist for thematic analysis provided by Braun and Clarke [33] was followed rigorously. In short, the process was firstly to read and reread the transcripts and comments. The next step was coding basic and meaningful units of information called coded elements (CEs). A total of 1723 CEs were then grouped into 231 basic categories or nodes. The categories were then reviewed and refined in a recursive process going back and forth to initial codes and evaluating their relation to the entire dataset. The coding was checked and agreed upon by both authors independently. Next, the level of abstraction was raised in which all nodes/categories were further grouped into eight overarching themes. The themes were then revised for consistency and finally named.

## Results

The analysis has revealed eight themes illustrating the content of the self-rapports and interactions of microdosers concerning (1) *microdosing motives, expectations, and context*; (2) *enhanced states and heightened senses*; (3) *insights and transformation*; (4) *improved abilities and optimal performance*; (5) *relief and cure for health conditions*; (6) *unwanted effects and lack of results*; (7) *microdosing approaches, strategies, and dosage*; and (8) *general viewpoints on microdosing.* The themes are presented below together with illustrative quotes. Since it is not accurate to label the individuals (unknowingly) providing the self-rapports of the present study for “respondents” or “participants,” we will for the readability of the text call them (the) “microdosers” or (the) “users” throughout.

### Microdosing motives, expectations, and context

Explicit motives to microdose were either a form of self-treatment (e.g., disorders), self-optimization (e.g., enhancing “normal” function), or a mix of both aspects. A tiny sample also mentioned exploratory reasons.

Microdosing was (in part) discussed as one of several modalities in a personal health-seeking endeavor, incorporating diet, exercise, meditation or other techniques for health, well-being, and personal development. A “holistic” approach to health was often premiered, and in this context, microdosing was viewed as a catalyst for improving the overall orientation and results of health- or self-optimization efforts. “I was already doing things to become less anxious and depressed, and to have more confidence. Things like yoga, meditation, eating right, working out, and doing personal development. But once I added mushrooms, it was like all of that, put on steroids.” Specific trends or lifestyle orientations (e.g., biohacking, personalized medicine, and transhumanism) were also mentioned in association with the interest in microdosing.

Enhancement incentives involved both physical and mental aspects and where sought by users with normal functioning, as well as in the context of limiting neuropsychiatric conditions. Sufferers of various illnesses sometimes viewed microdosing as a last-ditch effort to self-treatment when health care, prescribed treatments, or other methods were found insufficient. “I got more and more depressed, none of the medications were working, and eventually I became suicidal, and that’s when I thought I have nothing to lose, I might as well try something crazy.”

Not uncommonly, microdosers were motivated by previous high-dose psychedelic experiences and expressed a broad interest in psychedelics. Other users had no previous experiences of psychedelics or interest in psychoactive drugs and solely sought out microdosing as an effort to treat their conditions. “I really don’t like to get high.”

The interest in microdosing was sometimes said to be influenced by user stories and information from YouTube or other social media. The impact of the YouTube platform and how engagement in the microdosing trend is advanced were also demonstrated by the feedback from, and interactions with, the content viewers (commenters). Both critique and grateful appreciation of the videos, content creators, and their provided views and information were present throughout the data “Love your vids! Feels great to know others are on the same journey.” The comments section is often used to clarify information discussed in the videos and ask for advice regarding specific health issues or recommendations of additional information resources. “Great video and interview! When you quote the weight of a dose of shrooms are you quoting dry weight as in a powder?”

### Enhanced states and heightened senses

Microdosing was said to provide a subtle but significant change in psychophysiological states, effectively serving as an all-purpose or nonspecific amplifier. These (acute) effects appeared to be the foundation or prerequisite for the psychological advances and improved functioning outlined in the following three themes. As such, theme 3, 4, and 5 should be read as a progression or continuum proceeding from the more immediate change in states defined in this theme. However, some overlap and arbitrary cutoff between these four themes will inevitably occur. Also, the outlined progression might not, in reality, appear or manifest linearly on all occasions.

Most notably, the augmented states attributed to microdosing revolved around alterations of time perception and an increased (wider) sense of “now presence.” By being more present in the current moment, microdosers proclaimed to experience less mental chatter and more focus, un-disrupted engagement (flow), and perceptual clarity. “I was able to just “be” instead of constantly evaluating/analyzing/talking with myself about the way things are, and that’s a feeling that’s been elusive in my normal state.” The heightened sense of presence and perceptual clarity were said to mean a qualitative improvement in the experience of the present moment, both in the form of heightened visual acuity, increased awareness of sound, touch, and smell, but also in the affective quality of the subjective experience. The sensation of improved psychophysiological functioning was likened to “the expensive quality fuel high-performance cars require to run optimally.” Not uncommonly, users noted several enhancement effects concurrently. “Visual acuity goes off the charts. Same with hearing and physical performance. Leaves everything else in the dust!” Straightforward enhancement effects were also noted for mood, energy levels, and “drive.” “I felt this kind of like a bubbly sense of energy, this little sense of glow.” The effects on emotional states were further exemplified by a reduction in stress, sadness, anger, and other unwanted feelings. Also, increased patience, more openness, and a sense of groundedness and gratitude were mentioned as beneficial improvements in emotional states. Further psycho-spiritual changes are outlined in the following theme.

### Insights and transformation

A process of augmented self-reflection was often seen as central to the microdosing practice. The microdosers gave extensive descriptions of thoughtful insights and psycho-spiritual changes, reportedly enabling improvements in personal orientation, priorities, and habits. Primarily, the discussed insights and changes in proclivity appeared to proceed from an increased awareness of, and subsequent desire to, live “authentically” and with more connection to nature and care for the needs of self and others. “Yes, microdosing can be performance-enhancing, but it also increases the odds you’re going to do the kind of work that can, not only be of deeper alignment with yourself, but also work that will be of benefit to people beyond yourself.” A stronger sense of an authentic self and a clearer understanding of personal truths, or core beliefs, were often mentioned as a result of microdosing insights. “They’ve been able to show me who I am.”

One key component of transformational processes was reportedly how latent emotional content could be evoked by microdosing, providing an opportunity to recognize and process lingering trauma or to integrate a psychological “shadow” into more conscious levels of the psyche. “Microdosing revealed to me parts of my personality, my nature, that I wasn’t proud of and wasn’t easy to face.” Microdosers reported improved cognizance of a wide range of psychological, psychosocial, and lifestyle factors, indicating an increased capacity for emotional regulation and awareness of the impact of personal actions. Improvements in self-confidence and self-acceptance and a subsequent lessening of social unease were said to allow for an increased sense of empathy and deeper connections in personal relationships. Also, a more trusting and adaptive approach to the general progress or “flow of life” reportedly influenced well-being and quality of life positively for some users.

Microdosing was also discussed as a means of integrating previous insights and realizations attained by high-dose psychedelic use or other peak spiritual experiences. For these users, the microdosing practice was seen as a way to amalgamate a spiritual experience or practice (for instance, meditation) with the “awake state” and circumstances of everyday life. It was reasoned that today’s Western society does not premiere a more “pristine mode of being” and microdosers discussed realizations about the negative impact of living in a “disconnected culture and society.” “This world is plagued with junk food, porn, materialism, jealousy, and all these things. I think mushrooms connect us to a much simpler ancient way of viewing the world.” A sense of “reconnecting to nature” was reportedly an important and common transformational influence of microdosing. On the same note, it was described how the urge for unhealthy habits lessened significantly while the motivation for more exercise, healthier food, and less habitual use of social media was premiered. Also, users reported less procrastination and a spontaneous impulse to clean the house, tidy drawers, pay bills, or address other postponed or neglected tasks.

### Improved abilities and optimal performance

Ensuing from the previously described shifts in psychophysiological states and, to some extent, also the insights and transformations outlined in the previous theme, the microdosers reported a range of improved abilities or performance enhancement. Most commonly, increased access to creativity and enhanced productivity, including both convergent and divergent thinking, were discussed. Creative roadblocks or writer’s block was said to be overcome by augmented “out of the box thinking” and increased enjoyment of work. “Because you’re having an abundance of creative thoughts, it allows you to take a new spin on whatever work you’re doing, and it makes things more exciting.” Enhanced productivity and effectiveness were reported in tasks like software development, music composition, business management, academic work, and other cognitive intensive jobs and problem-solving. “I solve Rubik’s cubes, and when I did so on LSD, I could come up with way more efficient and quick solutions to certain cases.”

Athletic performance and exercising benefits of microdosing were exemplified by practitioners of many different sports and activities including ice hockey, basketball, freestyle climbing, MMA, and long-distance trail running. Increased energy, focus, coordination, prevision, and overall motivation were typical benefits attributed to the use of microdosing in sports and physical exercise. “I used to play every hockey game from 10th grade on LSD, and it really elevated my senses and instincts.”

A heightened sense of presence, extraversion, attendance, or persuasiveness in social situations reportedly improved the ability to influence others and generate new relations or work-related opportunities. “Sales meetings and marketing outreach was easier for me than it was before. When I tracked the metrics of performance, I performed about 20% better than without microdosing.” Increased leadership abilities with more adaptability and sensitivity to the needs and social feedback of coworkers were other mentioned benefits of microdosing in a professional setting.

### Relief and cure for health conditions

Several disorders or pathological functioning, not uncommonly resistant to previous efforts of curing, were reportedly alleviated or eased by microdosing. Most commonly, depression, also long-standing major depression and otherwise hard to treat cases, was said to be cured entirely or significantly eased. “Microdsoing LSD has been the best thing that happened in my life. Dealing with serious depression for 35 years… it’s now GONE!” A lessening of anxiety was also reported (however, these results appeared more contradictory, see theme six). Several microdosers stated how both depression and anxiety was lifted, and examples of liberation from catastrophizing thought patterns were presented. Freedom from dysfunctional beliefs and improved disease insight were attributed to microdosing by sufferers of various psychological issues. Individuals with PTSD and bipolar disorder reportedly experienced significant improvements. “I’m bipolar and used anti-depressives my entire life. The only medicine that works for me is Psilocybin mushrooms. I microdose and grow my own now. I have never felt better.”

Addiction to drugs (alcohol, narcotics, prescription drugs, and nicotine) was recurrently said to be effectively treated by a microdosing practice. “I quit smoking after 20 years. It’s like reprogramming your brain.” “It has also ended my alcoholism.” Microdosing was also said to assist with motivation and lifestyle changes post-addiction. “When I quit cannabis, my mind wound up in a very dark place. It wasn’t until I began microdosing psilocybin that I was able to do something positive for myself, both physically and mentally.”

Users with neuropsychiatric conditions (e.g., ADHD and ASD) noted improvements in cognitive and social abilities. “It can help people with Asperger’s learn to socialize without killing their love of science. At least that is what LSD and DMT did for me.” Relief from problematic stuttering was also attributed to microdosing in a few cases.

Accounts of what appeared to be neurological effects of microdosing were discussed in the context of paralysis and dyspraxia (a neurological motor coordination disorder). One case with paralysis from spinal cord injuries reported regained control of bladder function and also described peculiar leg spasms and movements when microdosing psilocybin mushrooms. “I’ve also regained my bladder control and bowel movement since my microdosing experiment started! It’s so amazing, I want to shout it from the mountain tops and tell everybody.” Dyspraxia symptoms were said to be successfully treated by microdosing in one case where prescribed medications were not perceived as appropriate or sufficient treatment. “Even microdosing once a month tends to alleviate dyspraxia symptoms. FDA leaves no options of medical treatment because they would rather prescribe me addictive anxiety medication instead of treating the actual diagnosis.” The intense desperation described by sufferers of treatment-resistant cluster headaches and how microdosing cured or lessened the frequency of pain episodes were other noteworthy testimonies of experienced health benefits.

### Unwanted effects and lack of results

Microdosing reportedly caused some adverse effects and discomfort, but no serious events or harm was reported. Also, mixed results or a lack of sought effects were noted. Most commonly, it appeared that a preferred “functional” microdose was not always established, and several examples of inadvertently dosing too high were reported. In some cases, microdosers found themselves noticeably affected while attending a workplace environment. However, this was typically discussed somewhat lightly as a comical or awkward occurrence, rather than a significant adverse event.

As mentioned in a previous theme, the effects on anxiety were sometimes complex or contradictory, and some users experienced increased anxiety, or occasionally even panic attacks, discouraging from further use. However, increased anxiety was also interpreted as a function of a therapeutic or cathartic process, where increased awareness initially intensified anxiety and stirred up negative emotions, and also provided more insights and possibilities to work through personal issues. “You do feel a higher degree of connectedness with yourself, to the point where if you do have anxious thoughts or if there’s something you’re worrying about it is possible for that worry to be enhanced.”

A sense of being physically uncomfortable or overly aware of bodily sensations sometimes discouraged further use. Gastrointestinal discomfort and cramping and increased body temperature were other sporadically reported unwanted effects. Also, sensations of restlessness and “jitters,” likened to the potentially unpleasant effects of caffeine, were mentioned and said to impact the ability to focus negatively. “Microdosing just made me hyper. It put me into a mental state of ‘do’ instead of ‘chill’, but at the same time, I become sloppy. Great if I just need to get off my arse to clean the house, but maybe not for solving complex problems or detailed work.” The over-stimulating effects were more associated with LSD than other substances. With its long-lasting effects, LSD was said sometimes to cause insomnia. Increased impulsivity appeared to be another related effect that could potentially mean unwanted outcomes. Exaggerated impulsivity was only mentioned in the context of long-standing microdosing practice over many months.

The pro-social and productivity-enhancing effects reported by microdosers were denied by a smaller sample experiencing contrary effects with increased introversion and a lessening of practical or problem-solving skills. It was speculated that incorrect (too high) dosage was the culprit of these outcomes. Decreased performance in specific cognitive domains while experiencing improvements in others—for example, less mechanical intelligence but improvements in social abilities—were also mentioned.

### Microdosing approaches, strategies, and dosage

Both the videos and comments were used to progress and exchange information regarding hands-on procedures, mental preparations, or other strategies for optimal results. Prevalent topics included dosage and administration, effect profiles of substances, and precautions to minimize risks or unwanted effects of microdosing.

The general aim of a preferred dose appeared to be subtle but noticeable (acute) effects, but not to impair or interfere with daily activities. A typical approach was to start with roughly 1/10th of a “full” or “recreational” dose every fourth day for a few weeks and then have a reset period before starting a new dosing period (i.e., the “Fadiman protocol” based on the recommendations from Fadiman [17]). It was also common that microdosers established (and recommend others to find) a dose and dosage interval according to personal needs and preferences. “Not daily though. Only when I feel I need a boost.” There were many examples of users finding the typical (1/10th) microdose either too palpable or, in other cases, wholly ineffectual and adjusted their dose accordingly. “I am very sensitive to psilocybin, it’s hard for me to accurately microdose.” The relationship between dosage and experiences of increased anxiety was discussed, and a strategy where the dosage was stepped up gradually, not to trigger anxiety, was suggested. “Microdosing causes extreme anxiety for me unless I take ‘a loading phase’ where I bring my tolerance up just a little and then resume to a regular microdose.” It was also proposed to handle anxiety by first using a “full dose” psychedelic treatment as an attempt to identify and work through any issues causing anxiety.

A small sample discussing entirely sub-perceptual doses for certain circumstances was also present. Typically, this approach combined a psilocybin microdose with other, non-psychoactive medicinal mushrooms and supplements for supposed epigenetic neurogenesis benefits as has been suggested by mycologist Paul Stamets.

Substances discussed were the classic serotonergic psychedelics, primarily LSD and psilocybin. However, analogs of both substances like 1P-LSD and 4-ACO-DMT were also common. Occasional mentions of other substances like *N*,*N*-DMT, ibogaine, mescaline, 2-CB, cannabis, and 5-MeO-DALT also occurred. The effect profiles of the most common substances, e.g., LSD and psilocybin, were said to not only overlap but also differ in several key ways. Besides being more long-lasting and with more energetic effects, LSD was said to be more tilted to benefit structured and focused cognitive work and psilocybin more to emotional awareness. Some preferred 4-ACO-DMT; claiming it provided both the cognitive clarity of LSD and the emotional awareness and groundedness of psilocybin.

To promote safety and avoid harm, it was recommended to first try a given substance and dosage at home on a non-workday. Other precautionary and practical recommendations included testing substances with a, for the purpose intended, reagent “test kit,” using a sensitive scale for correct doses, employ volumetric dosing (LSD) or grounding mushrooms to a powder before weighing the material for more predictability. It was also recommended to take breaks from microdosing to reset and avoid tolerance buildup and refrain from impulsive significant life decisions under the influence.

One prevailing sentiment regarding preparation and mindset was that intentions are a vital component to the effectiveness and potential benefits of microdosing. It was typically recommended to set clear intentions and to see microdosing as a tool to facilitate the realization of these objectives. On the same note, it was also advised to let go of specific expectations and trust the process. “Have clear and serious intent and be open to guidance and revelation.” It was considered preferable to keep some form of journaling of day-to-day status to monitor and recognize gradual changes over time. Performance- and athletics-oriented microdosers sometimes also utilized various self-tracking techniques, via biometrics or other objective measures to evaluate performance and avoid bias or misjudgments of microdosing effects. “For parasympathetic and sympathetic nervous system balance, you would want to test your heart rate variability.”

### General viewpoints on microdosing

This theme summarizes the general viewpoints on microdosing discussed by users; what microdosing is, why and how it works, and how it compares to other methods or medications were prevalently deliberated topics. Discussions on presumed mechanisms of action typically included both biochemical and psycho-spiritual explanations. Scientific concepts like neurogenesis and neuroplasticity were attributed to the sense of heightened adaptability and benefits on cognitive functioning. Improved mood, confidence, and posture were credited to increased serotonin activity. Microdosers described how these supposed biological changes, guided by their intentions and efforts, allowed for a sort of “reprogramming of the mind-body interface.” Also, more esoteric or mystical concepts like the presence of an intrinsic “intelligence” or “spirit of the mushroom” and certain “homing” properties, where the mushrooms somehow impact the specific issues most needed of a user, were sometimes speculated. Some conceptualized the benefits of a microdosing practice as a “spiritual repair,” and others saw it as a “psychotherapeutic tool.”

A key perspective was that psychedelics fundamentally differs from established or prescribed psychiatric treatment in the sense that they do not mask issues or facilitate a passive coping but instead can assist the user in recognizing the root of their problems and working through them. “Instead of blunting our emotional response to trauma that we’ve had in the past, the trauma starts to come up, and we have to look at it and integrate it in a way that helps us to become a more healthy self.” The effect profile of microdosing was similarly compared to and sometimes favored over prescription stimulants (ADHD medication), as well as off-label stimulants employed for exercising purposes. “Adderall has this feeling of like forcing you to focus, whereas, with LSD, it’s more like you want to.” Microdosing was also sometimes preferred over high doses of psychedelic substances as less intense and tasking, but still tangible process through more gradual changes over time. “It gives the mushroom time to work in your system. I find with anything that you take more consistently but moderately, that you give it time to work deeper into your body.”

Provided microdosing was used in a purposeful and structured way; it was generally understood to be a relatively safe and affordable intervention with low abuse-potential and few serious side effects*.* Many users viewed microdosing as immensely beneficial, and several testimonies of how microdosing had “saved my life” and steered away from suicide were presented. Others concluded that microdosing had certain benefits, but exercise, meditation, and other lifestyle factors were overall more critical. One of the most significant negatives discussed was the illegality status of most substances used, and prescription access to microdosing was seen as much preferable to the current situation. Some users expressed a sense of embarrassment and stigma for taking part in illegal methods.

## Discussion

This study provides a systematic qualitative analysis of psychedelic microdosing in a natural setting, as it is discussed and characterized online via the YouTube.com platform. The videos containing self-rapports were often longer format-free form discussions or single-person video blogging, and together with the subsequent comments section, they provided in-depth and rich qualitative data on the motivations, expectations, effects, perceived mechanisms of action, and outcomes experienced by microdosers. Also, the results offer some insight into how microdosing is conducted, contextualized, and communicated among users. Microdosing motives, reported effects, and context were partly in line with the trending (media) narrative of microdosing as a “productivity hack” or performance enhancer, but the reports of the present study were more tilted to various forms of therapeutic and self-reflective (spiritual) uses. Not rarely, there was also an overlap between self-treating and self-optimization incentives, and many users valued multiple aspects of microdosing. A small subset of users mentioned exploratory reasons (curiosity), but while the effects of microdosing sometimes were said to be enjoyable, it was not discussed as a recreational activity.

Typically microdosing was performed in a cyclic and semi-structured regimen with the intent of personal improvements, transformation, or health. LSD and psilocybin were the most common substances, but other more novel psychedelics were also used according to preference and availability. Primarily, positive or beneficial effects and fewer, mostly minor, side effects were reported. However, some users mentioned a lack of sought after, or even contrary, effects, like reduced performance for specific tasks. Possible long-term risks were considered in a few videos, but no self-rapports of harm from long-term use were presented.

Therapeutic effects on depression appeared especially noteworthy, and reports were unanimously positive, but other mental health-related issues like trauma, addiction, bipolar disorder, and anxiety were also said to be improved or fully resolved through microdosing. Interestingly, reports involving anxiety were more disparate than for all other indications, whereas users also experienced increased anxiety and negative emotions. In the microdosing study by Polito and Stevenson [14], a similar undesirable effect of increased neuroticism was identified. One explanation can be that anti-amnesic or revealing properties of psychedelic substances [34] are hypothetically also present at low doses. Increased cognize of troubling sides of personal or other circumstances could understandably mean elevated anxiety.

The claimed beneficial microdosing effects can seem unrealistically broad ranging. On the other hand, evidence from full-dose psychedelic research shows a comparable width of beneficial results. For instance, research has provided evidence for the effectiveness of psychedelic substances in treating addiction, depression, and PTSD [e.g., [6, 35, 36]]. Also, our findings parallel with positive psychology research of full-dose psychedelic use where acute and lasting effects on mood, well-being, prosocial behaviors, cognitive flexibility, creativity, value alignments, nature-connection, personality factor openness, and mindfulness-associated capabilities have been shown [37].

Most of our findings regarding motivations and applications for microdosing have been addressed by at least one previous study, but to our knowledge, a few approaches and outcomes have not been discussed before. One of which was the integration and incorporation of insights from spiritual practices (meditation), high-dose psychedelic experiences, or other peak experiences into the “normal state” of everyday life. Interactions with the everyday milieu in a slightly “enhanced state” were claimed to facilitate the implementation of new and preferable ways of being, otherwise compartmentalized or only accessible in significantly altered states. Additionally, a smaller sample reported improvements for dyspraxia and paralysis (from spinal cord injuries) not previously discussed in any microdosing study. This sample stated other unrelated intentions for microdosing and noted the curative effects seemingly surprised.

Other noteworthy uses for microdosing were self-treatment for neuropsychiatric conditions, cluster headaches, and displacement of prescription drugs. Individuals diagnosed with ADHD or ASD reported expedient effects from microdosing and sometimes replaced Ritalin or Adderall with a low-dose psychedelic substance. Interestingly, the maker of LSD, Dr. Albert Hoffman, proposed small doses of LSD as a suitable alternative to Ritalin with a preferable effect profile [17]. Microdosing reportedly also functioned as a replacement for other prescription drugs viewed to have inferior effect profiles (for instance, SSRIs). Previous studies have indicated successful self-treatment of headache disorders with psychedelics and microdosing [24, 38, 39], and also, in the present study, microdosing was claimed an effective strategy in lessening the intensity and frequency of cluster headaches.

The method and dataset (self-rapports) used for this study can only provide rough estimations of dosages used. Furthermore, we found the commonly suggested starting point of 1/10th of a “recreational” or “full” dose was often deemed either too much for daily activities or completely ineffectual, indicating high sensitivity variability among users or potency and amount of the ingested material. Also, it appears both from the rapports of this study together with early clinical data of low-dose LSD responses [18] that the “window” between no perceptible effects and where effects start to adversely interfere with cognitive functioning and regular day-to-day activities most likely are very narrow.

Nevertheless, based on the qualitative insights of the present study, we suggest microdosing definitions tied to fixed dose ranges [23] might be redundant, or at least too individually varying to be particularly useful to understand the actual microdosing practice or phenomenon as it occurs, which appears more a matter of *how* the psychedelic substance is used. Here, the definition of microdosing is clearer; in practice, we identify microdosing to generally mean recurrent but spaced doses of a psychedelic substance, small enough to not impede normal daily activities but large enough to provide subtle noticeable acute effects. Also, it appeared from the rapports that microdosing rarely occurred without explicit and direct (typically intimate) intentions for use, and this was commonly viewed as key to the full potential and the transformative processes ascribed to microdosing. “On its own, it is not going to ‘help’ you necessarily, other than a shift in mood. If you microdose and sit around playing video games, it won’t change your life. If you do it and focus on shifting yourself into a new reality, then I think it can help facilitate that by making your mind more receptive to change and allowing you to notice new things about yourself that you might not have otherwise”.

Microdosing was often incorporated alongside other modalities or lifestyle factors for health or well-being and was said to support and better align the orientation of these efforts. Although microdosing was sometimes strongly advocated, it was not typically discussed as an effortless panacea, but more enabling the user to “get the ball rolling” and ease the process of implementing needed changes, while also making appropriate changes more apparent.

The facilitation of self-reflection and personal insights appeared equally valued by healthy individuals and those combating mental health issues. However, the taxonomy of “healthy individuals” was challenged by the idea that most individuals carry a degree of traumas and fears impacting function and quality of life, and microdosing was said to facilitate awareness and transcendence of these traumatic “imprints.” Similar observations of the overlap or lack of clear distinction between therapy and enhancement usage and a questioning of the concept of “healthy” designated to undiagnosed individuals were interestingly also noted by respondents in the Johnstad study [16].

The sample appeared to be relatively diverse (prior knowledge, drug experience, age, gender, motives), but one commonality was a willingness to take on responsibility for personal health and development, either by a general lifestyle outlook or sheer desperation from not finding prescribed treatments and available healthcare sufficient. The social evolvement where people increasingly turn to health information and support groups on the internet and a lessening of the cultural authority of medical experts are noted and addressed in studies from various academic disciplines [e.g., [40–42]] our drug discussion studies [24] and prior microdosing studies [16]. Also, discontent with conventional therapies and adverse effects can incentivize the use of complementary and alternative medicine (CAM) [43]. The use of CAM has seen a rise over the last decades in both the USA and Europe [43, 44]. At least in part, the displacement trend towards health information-seeking behavior, CAM usage, and Internet support groups (ISG) can help to contextualize the growing microdosing phenomenon.

Our examination of the comments following the videos revealed several peer-to-peer support aspects and how a joint creation and exchange of information regarding microdosing is advanced. The conversations between users (and those interested in microdosing) covered practical information regarding dosage, substances, safety precautions, mental preparations, and strategies for optimal results. Another function of interactions was to comment or correct various factual claims in the videos and ask for clarifications and additional information as well as sharing of social support and feedback. Enthusiastic claims of microdosing benefits were prevalent, but overall, the discussions were reasonably nuanced, risk-aware, and appeared to generally be derived from an intent of personal and mutual betterment. The YouTube platform was also referenced as a source for information initially producing a personal interest in microdosing, which highlights the impact of the platform on the proliferation of microdosing practices.

Our findings were overall in agreement with previous microdosing studies [13–17], but at present, there is no way to discern reported outcomes from placebo or how expectations or other lifestyle factors and interventions influence outcomes. In our view, it is likely that expectations may impact both negative and positive outcomes. However, considering psychedelic substances appear to be physiologically active at microdosing ranges [18], and the various mechanisms of action known from psychedelic neuroscience [37], the overall credibility of the reported outcomes do not seem unlikely in principle. Potentially, microdosing could provide some of the same benefits (for certain conditions) of full dose interventions with less risk of adverse reactions related to the sometimes intense experiences of higher doses. Microdosing could also mean additional benefits, as well as risks, through the repeated exposure over extended periods. Also, considering how the use typically takes place in an “everyday context” while interacting with acquainted work-related tasks and personal relations, new applications and benefits not attainable by the occasional and drastically altered states of full doses are plausible.

## Limitations, strengths, and future research

This study used a new and novel methodology to obtain and process research data from YouTube, and therefore, we have taken extra care to describe the approach in full detail. Naturally, there is room for improvements or modifications, and to fully comprehend potential usefulness of the method, it should be evaluated further and applied to additional research projects. So far, the approach appears to be efficacious and shows potential to be expedient in future research. A challenge of the method is the vast amount of data a topic can generate and how to sort, discern, and present relevant information in a consistent way.

We consider a primary strength of the study to be the broad and truly inductive approach and the absence of preconceived survey questions or other researcher constructs. An apparent limitation of this study is the difficulty in controlling the conditions surrounding the drug use (e.g., dosage, type of substance, combinations with other drugs or medications). The self-selected and non-randomized sample could contribute to bias, and the accuracy of reports cannot be guaranteed. The reported therapeutic outcomes can be questioned as selective reporting or incorrect recall. However, if lop-sided reporting was the reason for the robust results on depression, we suggest that the same would be presented for anxiety, where treatment results were highly contradictory.

We also recognize it is not likely a generalizable sample publishing self-disclosure videos of illegal drug use on YouTube. However, a predominant part of self-rapports used for the present study was derived from the comments sections where users can be fully anonymous and are not likely to be censored or reprimanded for such disclosure. Also, considering the general nature of YouTube usage and the videos were posted over eight separate YouTube categories, by 25 channels, with a total of 4.5 million subscribers, we suggest the present sample consists of a less “specialized” demographics than in any available drug discussion forum.

For future clinical research, we propose the importance of incorporating the variable of personal intentions. If microdosing primarily has the most useful function as a tool to realize personal intentions that variable should be carefully considered. The narrow and individually varying dose window of a psychedelic microdose noted in the present study is also essential to take into consideration. Overlooking these factors risks producing skewed results where potentially essential outcomes, otherwise present in a context where the microdoser self-corrects the dose and frequency according to personal preference and situation, can be overlooked.

## Conclusions

Microdosing with various psychedelic substances, primarily LSD and psilocybin, was used for therapeutic and enhancement purposes and mostly beneficial effects were reported. Therapeutic effects for depression appeared especially noteworthy. Many different applications and outcomes were discussed, and the function of interactions was primarily to exchange views on the microdosing phenomenon, progress strategies for optimal results, minimize risks, and share social support. Therapeutic outcomes appeared to be supported by microdosing in a self-reinforcing process: (1) motivation to handle issues was raised by immediate increases in mood, energy, presence, and attendance; (2) awareness of problems and solutions was raised by access to new perspectives, a sense of reconnecting to an authentic self, and core beliefs; and (3) change of habits and mindset was eased by increased affinity for healthy lifestyle factors and an augmented sense of adaptability. Intentions for use were recognized as an important factor for the outcomes of microdosing. The current trend of microdosing and its claimed health benefits are highly interesting, but questions of long-term risks are still unanswered, and the potential benefits request more evidence.

## Data Availability

For ethical reasons, the dataset will only be submitted upon individual requests.

## Abbreviations

ADHD: Attention deficit disorder
API key: Application programming interface key
ASD: Autism spectrum disorder
CAM: Complementary and alternative medicine
CE: Coded elements
DMT: *N*,*N*-dimethyltryptamine
HPPD: Hallucinogen-persisting perception disorder
ISG: Internet support groups
LSD: Lysergic acid diethylamide
MMA: Mixed martial arts
PTSD: Post-traumatic stress disorder
SSRI: Selective serotonin reuptake inhibitor
